# Molecular and clinical analyses of 84 patients with tuberous sclerosis complex

**DOI:** 10.1186/1471-2350-7-72

**Published:** 2006-09-18

**Authors:** Chia-Cheng Hung, Yi-Ning Su, Shu-Chin Chien, Horng-Huei Liou, Chih-Chuan Chen, Pau-Chung Chen, Chia-Jung Hsieh, Chih-Ping Chen, Wang-Tso Lee, Win-Li Lin, Chien-Nan Lee

**Affiliations:** 1 Institute of Biomedical Engineering, College of Medicine and College of Engineering, National Taiwan University, Taipei, Taiwan; 2 Department of Medical Genetics, National Taiwan University Hospital, Taipei, Taiwan; 3 Departments of Medical Genetics and Obstetrics and Gynecology, China Medical University Hospital, Taichung, Taiwan; 4 Department of Neurology, National Taiwan University Hospital, Taipei, Taiwan; 5 Department of Pharmacology, College of Medicine, National Taiwan University, Taipei, Taiwan; 6 Institute of Occupational Medicine and Industrial Hygiene, National Taiwan University College of Public Health, Taipei, Taiwan; 7 Department of Obstetrics and Gynecology, Mackay Memorial Hospital, Taipei, Taiwan; 8 Department of Pediatrics, National Taiwan University Hospital, Taipei, Taiwan; 9 Department of Obstetrics and Gynecology, National Taiwan University Hospital, Taipei, Taiwan; 10 Graduate Institute of Clinical Medicine, College of Medicine, National Taiwan University, Taipei, Taiwan

## Abstract

**Background:**

Tuberous sclerosis complex (TSC) is an autosomal dominant disease characterized by the development of multiple hamartomas in many internal organs. Mutations in either one of 2 genes, *TSC1 *and *TSC2*, have been attributed to the development of TSC. More than two-thirds of TSC patients are sporadic cases, and a wide variety of mutations in the coding region of the *TSC1 *and *TSC2 *genes have been reported.

**Methods:**

Mutational analysis of *TSC1 *and *TSC2 *genes was performed in 84 Taiwanese TSC families using denaturing high-performance liquid chromatography (DHPLC) and direct sequencing.

**Results:**

Mutations were identified in a total of 64 (76 %) cases, including 9 *TSC1 *mutations (7 sporadic and 2 familial cases) and 55 *TSC2 *mutations (47 sporadic and 8 familial cases). Thirty-one of the 64 mutations found have not been described previously. The phenotype association is consistent with findings from other large studies, showing that disease resulting from mutations to *TSC1 *is less severe than disease due to *TSC2 *mutation.

**Conclusion:**

This study provides a representative picture of the distribution of mutations of the *TSC1 *and *TSC2 *genes in clinically ascertained TSC cases in the Taiwanese population. Although nearly half of the mutations identified were novel, the kinds and distribution of mutation were not different in this population compared to that seen in larger European and American studies.

## Background

Tuberous sclerosis complex (TSC) is an autosomal dominant disorder having an incidence of 1 in 6,000 to 1 in 10,000 live births [1]. The severity of TSC and its impact on the quality of life are extremely variable among patients [2]. Common clinical manifestations of this disease include intellectual handicap, autistic disorders, and epilepsy due to the frequent, widespread occurrence of cortical tubers, which are focal disruptions of the cortical architecture due to undifferentiated giant cells. Hamartomas are also found in multiple other organ systems, including the heart, lungs, kidneys, and skin [3].

Patients often seek medical attention for dermal lesions or frequent seizures. The clinical diagnostic guidelines on TSC were prepared based on clinical features, radiographic findings, and histopathological findings [3]. Accurate clinical diagnoses are relatively easy in patients with classic multisystem involvement, but are often difficult due to the diversity of clinical findings in TSC patients.

The genetic basis of TSC has been determined to be due to mutation in either one of two unlinked genes, *TSC1 *and *TSC2 *[4]. The human *TSC1 *gene on chromosome 9q34 consists of 23 exons giving an 8.6-kb mRNA transcript, which has a coding region of 3.5-kb and encodes a 130-kDa protein spanning 1164 amino acids [5]. The *TSC2 *gene, which is located on chromosome 16p13.3, contains 41 exons and encodes a 200-kDa protein with 1807 amino acid [4,6]. Both *TSC1 *and *TSC2 *are tumor suppressor genes and their protein products, hamartin and tuberin, respectively, form a complex that regulates the mammalian target of rapamycin (mTOR) in the phosphoinositide 3-kinases (PI3-kinase)/AKT pathway to control cellular proliferation, adhesion, growth, differentiation or migration [7,8]. Furthermore, both genes play a role in cortical differentiation and growth control.

The mutation spectra of the *TSC *genes are very heterogeneous and no hotspots for mutations have been reported. There are many mutations in each gene that are seen recurrently, but no single mutation accounts for more than about 1% of all TSC patients. *TSC2 *mutations are about five times more common than *TSC1 *mutations [9] and new mutations are typically found in the two-thirds of TSC cases that are sporadic [10]. Despite complete penetrance of the disease in TSC patients, phenotypic variability can make the determination of disease status difficult among family members of affected individuals.

In this study, we analyzed both *TSC1 *and *TSC2 *genes in 84 independent Taiwanese TSC probands for whom detailed information on clinical manifestations and phenotype were available. Furthermore, we also assessed the mutational distribution and possible genotype-phenotype correlations between and within the two genes.

## Methods

### Patient Population

This study was approved by the Ethics Committee of the Division of Obstetrics and Gynecology, National Taiwan University Hospital. Eighty-four unrelated patients with confirmed clinical diagnoses of TSC and their family members were tested for mutations in *TSC1 *and *TSC2 *genes.

The general clinical features of TSC patients were determined by clinicians in accordance with the TSC diagnosis criteria set forth by the Tuberous Sclerosis Consensus Conference [3]. All patients' symptoms were investigated by a person blind to mutational status. High-resolution brain magnetic-resonance imaging (MRI) or computed tomography (CT) was performed on most patients.

The extent of facial angiofibroma or forehead plaques, non-traumatic ungal or periungal fibromas, hypomelanotic macules, shagreen patches, multiple retinal nodular hamartomas, cortical tubers, subependymal nodules, subependymal giant cell astrocytomas, cardiac rhabdomyomas, lymphangiomyomatoses, renal angiomyolipomas and confetti-like lesions were all assessed. Moreover, most patients' medical histories of mental development were assessed by a certified psychologist.

### Sample Preparation

After genetic counseling and obtaining informed consent, 5–10 mL of peripheral blood were collected from the participants. Genomic DNA was isolated from peripheral whole blood using the Puregene DNA Isolation Kit (Gentra Systems, Inc., Minneapolis, MN, USA).

### Mutational Analysis of *TSC *Genes

PCR primers and running conditions for each exon were available from previous studies [11-13]. The PCR reaction was run on each exon with a total sample volume of 25 μL containing 100 ng of genomic DNA, 0.12 μM of each respective primer, 100 μM dNTPs, 10 mM Tris-HCl (pH 8.3), 50 mM KCl, 2 mM MgCl_2_, and 0.5 units of AmpliTaq Gold enzyme (PE Applied Biosystems, Foster City, CA, USA). Amplification was performed in a multiblock system thermocycler (ThermoHybaid, Ashford, UK). The PCR amplification started with a denaturing step at 95°C for 5 minutes, followed by 35 cycles of denaturing at 94°C for 30 seconds, annealing at melting temperature (Tm) for 30 seconds, extension at 72°C for 45 seconds, and ends with a final extension step at 72°C for 10 minutes.

The screening of mutations was performed using the Transgenomic Wave Nucleic Acid Fragment Analysis System (Transgenomic Inc, San Jose, CA) with a C_18 _reversed-phase column containing 2-μm nonporous poly (styrene/divinylbenzene) particles (DNASep Column, Transgenomic Inc). PCR products were analyzed using linear acetonitrile gradients and triethylammonium acetate acting as mobile phases with the provision of buffer A (0.1 M TEAA) and buffer B (0.1 M TEAA with 25% acetonitrile) (WAVE Optimized, Transgenomic Inc). Heteroduplex analyses were performed according to the manufacturer's protocol and of previous studies [14,15].

### Statistical method

The χ^2 ^and Fisher exact tests were used to examine the differences in clinical manifestations, phenotypes, and mutation distributions in independent Taiwanese probands between patients with *TSC1 *and *TSC2 *genes.

### Direct Sequence Analysis

PCR products were purified by solid-phase extraction and bidirectionally sequenced using Applied Biosystems' Taq DyeDeoxy terminator cycle sequencing kit (Applied Biosystems). Sequencing reactions were separated on a PE Biosystems 373A/3100 sequencer.

## Results and Discussion

### Identification and Characterization of Mutations

In the current study, we performed mutational analysis on the coding exons and the exon/intron junctions of both *TSC1 *and *TSC2 *in a total of 84 individuals with TSC and their family members. The determination of mutation vs. polymorphism was done by: 1) checking the mutation tables at the Chromium site (); 2) comparison of findings to those of 100 healthy Taiwanese controls; and 3) checking the families similarly.

Nine mutations were identified in the *TSC1 *gene while 55 were identified in the *TSC2 *gene. Mutations in the *TSC1 *gene included five nonsense mutations with early termination codons and four insertions/deletions which caused frameshifts and resulted in premature truncation of the protein. Three of these mutations were novel, while six were previously reported (Table 1).

**Table 1:** Status of *TSC1 *mutations in Taiwanese patients with TSC

**No**.	**Gene**	**Exon**	**Nucleotide change**	**Codon change**	**Mutation type**	**Inheritance**	**Reported**	**Reference**
62	TSC1	7	c.602_604del CCT		In-frame deletion	S	N	This study
61	TSC1	15	c.1525C>T	p.R509X	Nonsense	F	R	[5]
72	TSC1	15	c.1791_1792dupAA		Frameshift	S	N	This study
2	TSC1	15	c.1884_1887delAAAG		Frameshift	F	R	[5]
36	TSC1	15	c.1959dupA		Frameshift	S	R	LOVD*
54	TSC1	17	c.2074C>T	p.R692X	Nonsense	S	R	[5]
31	TSC1	18	c.2283C>A	p.Y761X	Nonsense	S	R	[24]
3	TSC1	18	c.2332C>T	p.Q778X	Nonsense	S	N	This study
41	TSC1	18	c.2356C>T	p.R786X	Nonsense	S	R	[5]

Total: 9, F:2, S:7, N:3, R:6 MM:0, NM:5, FM:4, SM:0.								

F: familial case, S:sporadic case.

N: non-reported, R: reported.

MM: missense mutations, NM: nonsense mutations, FM: frameshift/in-frame mutations, SM: splicing site mutations.

* The the Leiden Open (source) Variation Database which was available at

The 55 mutations in the *TSC2 *gene included 12 missense, 15 nonsense, 21 frameshifts due to insertions and deletions and 7 putative splice-site mutations. Twenty-seven of these mutations were previously reported while 28 were novel (Table 2). Of the familial *TSC2 *missense mutations, A1141T and R1793Q may be rare polymorphic variants co-segregating with TSC. There was no direct evidence that these familial *TSC2 *missense mutational changes were pathogenic.

**Table 2:** Status of *TSC2 *mutations in Taiwanese patients with TSC

**No**.	**Gene**	**Exon**	**Nucleotide change**	**Codon change**	**Mutation type**	**Inheritance**	**Reported**	**Reference**
21	TSC2	1	c.109dupG		Frameshift	F	N	This study
30	TSC2	1	c.133_136delCTGA		Frameshift	S	R	DK*
35	TSC2	3	c.268C>T	p.Q90X	Nonsense	S	R	[25]
47	TSC2	6	c.632delC		Frameshift	S	N	This study
8	TSC2	intron 8	c.848+3delG		Splicing	S	N	This study
37	TSC2	9	c.856A>G	p.M286V	Missense	F	R	[10]
78	TSC2	10	c.1060C>T	p.Q354X	Nonsense	S	N	This study
75	TSC2	10	c.1117C>T	p.Q373X	Nonsense	S	R	DK*
48	TSC2	11	c.1226_1230delAACTG		Frameshift	S	N	This study
12	TSC2	12	c.1336C>T	p.Q446X	Nonsense	S	R	[25]
20	TSC2	14	c.1513C>T	p.R505X	Nonsense	S	R	[10]
57	TSC2	14	c.1513C>T	p.R505X	Nonsense	S	R	[10]
65	TSC2	intron 14	c.1599+2T>C		Splicing	S	N	This study
76	TSC2	16	c.1794C>G	p.Y598X	Nonsense	S	R	[10]
29	TSC2	16	c.1832G>A	R611Q	Missense	S	R	[10]
59	TSC2	intron 16	c.1840-2A>T		Spilicing	S	N	This study
82	TSC2	17	c.1939G>A	p.D647N	Missense	S	R	[26]
7	TSC2	18	c.2086T>C	p.C696R	Missense	S	R	[27]
53	TSC2	19	c.2103_2105dupTGA		In-frame insertion	S	N	This study
5	TSC2	19	c.2210T>C	p.L737P	Missense	S	N	This study
23	TSC2	20	c.2251C>T	p.R751X	Nonsense	S	R	[10]
70	TSC2	20	c.2251C>T	p.R751X	Nonsense	S	R	[10]
39	TSC2	21	c.2404dupA		Frameshift	F	N	This study
32	TSC2	21	c.2461A>T	p.K821X	Nonsense	S	N	This study
11	TSC2	21	c.2538delC		Frameshift	F	N	This study
67	TSC2	intron 21	c.2546-2A>T		Splicing	S	N	This study
73	TSC2	intron 22	c.2639+1G>C		Splicing	S	R	[9]
22	TSC2	23	c.2641delT		Frameshift	F	N	This study
27	TSC2	24	c.2824G>T	p.Q942X	Nonsense	S	N	This study
64	TSC2	26	c.2974C>T	p.Q992X	Nonsense	S	R	[28]
80	TSC2	26	c.3076dupT		Frameshift	S	N	This study
33	TSC2	28	c.3389delC		Frameshift	S	N	This study
19	TSC2	29	c.3412C>T	p.R1138X	Nonsense	S	R	[9]
42	TSC2	29	c.3421G>A	p.A1141T	Missense	F	N	This study
13	TSC2	30	c.3693_3696delGTCT		Frameshift	S	R	DK*
51	TSC2	30	c.3696dupT		Frameshift	S	N	This study
9	TSC2	33	c.4175_4176delAG		Frameshift	S	N	This study
26	TSC2	33	c.4440dupA		Frameshift	S	N	This study
77	TSC2	34	c.4541_4544delCAAA		Frameshift	S	R	[12]
18	TSC2	35	c.4603_4605delGAC		In-frame deletion	S	N	This study
34	TSC2	35	c.4603G>T	p.D1535Y	Missense	S	N	This study
83	TSC2	36	c.4830G>A	p.W1610X	Nonsense	S	R	DK*
28	TSC2	36	c.4846C>T	p.Q1616X	Nonsense	S	N	This study
16	TSC2	37	c.4909_4910delAA		Frameshift	S	N	This study
81	TSC2	38	c.5032dupT		Frameshift	S	N	This study
60	TSC2	39	c.5150T>C	p.L1717P	Missense	S	R	[29]
55	TSC2	intron 39	c.5160+3G>C		Splicing	S	N	This study
43	TSC2	intron 39	c.5160+4A>G		Splicing	S	R	[29]
4	TSC2	40	c.5227C>T	p.R1743W	Missense	S	R	DK *
50	TSC2	40	c.5227C>T	p.R1743W	Missense	S	R	DK*
56	TSC2	40	c.5228G>A	p.R1743Q	Missense	F	R	[30]
10	TSC2	40	c.5238_5255del18		Frameshift	S	R	[31]
25	TSC2	40	c.5238_5255del18		Frameshift	S	R	[31]
6	TSC2	40	c.5252_5259+19del27		Frameshift	S	R	[9]
15	TSC2	41	c.5378G>A	p.R1793Q	Missense	F	N	This study

Total: 55, F:8, S:47, N:28, R:27 MM:12, NM:15, FM:21, SM:7.								

F: familial case, S:sporadic case.

N: non-reported, R: reported.

MM: missense mutations, NM: nonsense mutations, FM: frameshift/in-frame mutations, SM: splicing site mutations.

* The database of Dr David Kwiatkowski which was available at

For both genes, sequence variants that were possible mutations were tested in all other family members, including the parents and both the affected and the unaffected family members. In total, 31 of the 64 mutations (48%) had not been reported elsewhere. Moreover, no mutational hotspots were identified in either gene, with only four different mutations being found twice in TSC2.

Compared with those of European and American counterparts [9,10,16], the distribution of the *TSC1 *and *TSC2 *mutations among Taiwanese population is similar. Therefore, the spectrum of mutations seen among the Taiwanese is no different in comparison to those already reported thus far for these two genes, based on the genetic analyses of European and American TSC patients using the Fisher exact test (*P *= 0.85, 0.46, and 0.14, respectively).

### Identification and Characterization of Polymorphism

In order to identify whether the observed changes were mutations or polymorphisms, samples from 100 normal individuals serving as controls were analyzed. Changes that were not found in more than 200 control alleles were considered pathogenic. Therefore, unique or less frequent changes such as missense and splicing site mutations (Table 2) were considered likely pathogenic mutations. The nonpathogenic *TSC1 *and *TSC2 *mutations identified in the Taiwanese TSC patients are described in Table 3. We identified nine nonpathogenic polymorphisms in the *TSC1 *gene and 12 in the *TSC2 *gene. The nonpathogenic sequence variants were identified in both the TSC patients and the normal controls. Fourteen of these polymorphisms had not been reported previously (4 at the *TSC1 *locus and 10 at the *TSC2 *locus) that included one missense variant within the *TSC1 *coding region.

**Table 3:** Polymorphisms identified for *TSC1 *and *TSC2 *in Taiwanese TSC population.

**TSC1**						
**Exon**	**Nucleotide change**	**Codon change**	**Polymorphism type**	**Frequency**	**Reported**	**Reference**

Intron 3	c.106+15		Intron	13 (16 %)	N	This study
10	c.965 T>C	p.M322T	Missense	9 (11%)	R	[24]
Intron 11	c.1142-33 A>G		Intron	9 (11%)	R	LOVD^a^
Intron 12	c.1264-12 T>C		Intron	3 (4 %)	N	This study
Intron 14	c.1437-37 C>T		Intron	9 (11%)	R	LOVD^a^
15	c.1726 T>C	p.L576L	Silent	11 (13 %)	N	This study
15	c.1960 C>G	p.Q654E	Missense	3 (4 %)	N	This study
Intron 18	c.2392-35 T>C		Intron	9 (11%)	R	[24]
22	c.2829 C>T	p.A943A	Silent	3 (4 %)	R	[24]

**TSC2**						

**Exon**	**Nucleotide change**	**Codon change**	**Polymorphism type**	**Frequency**	**Reported**	**Reference**

14	c.1593 C>T	p.I531I	Silent	3 (4 %)	R	[26]
Intron 15	c.1717-30 G>A		Intron	2 (2 %)	N	This study
Intron 15	c.1717-27 G>A		Intron	1 (1 %)	N	This study
Intron 21	c.2545+45 T>A		Intron	11 (13 %)	N	This study
23	c.2652 C>T	p.Y884Y	Silent	1 (1 %)	N	This study
26	c.3126 G>T	p.P1042P	Silent	1 (1 %)	R	DK^b^
Intron 27	c.3285-19 C>T		Intron	1 (1 %)	N	This study
29	c.3475 C>T	p.R1159R	Silent	1 (1 %)	N	This study
33	c.4047 G>A	p.A1349A	Silent	2 (2 %)	N	This study
Intron 33	c.4493+18 G>A		Intron	1 (1 %)	N	This study
Intron 38	c.5069-21 G>A		Intron	1 (1 %)	N	This study
Intron 39	c.5161-9 C>T		Intron	7 (8 %)	N	This study

* Frequence means the number of cases in 84 Taiwanese TSC patients.

a The the Leiden Open (source) Variation Database which was available at

b The database of Dr David Kwiatkowski which was available at

### Genotype-Phenotype Correlation: Familial or Sporadic TSC mutations

**Figure 1:**
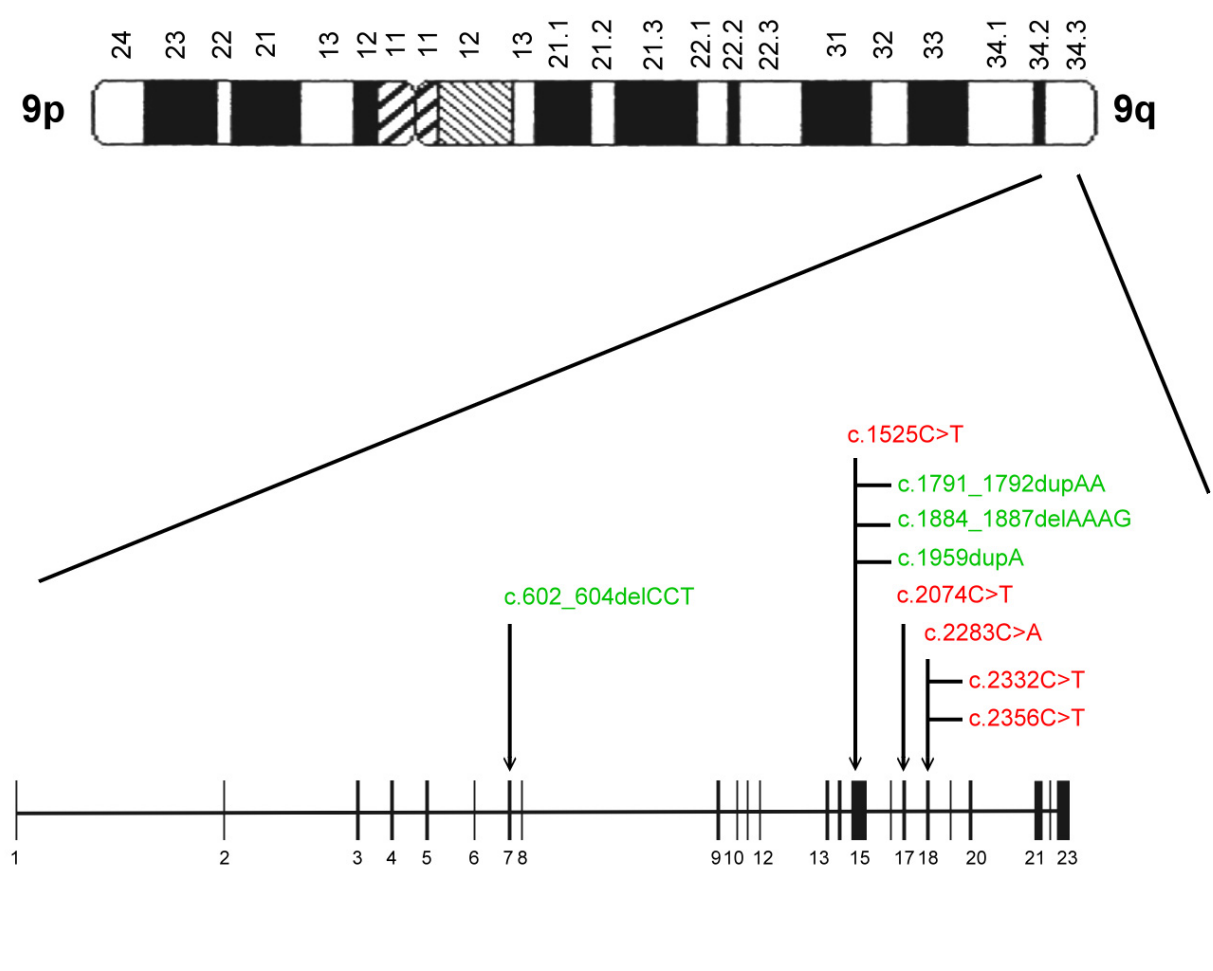
Diagram depicting the locations of mutations in the *TSC1 *gene. Nonsense (*red*), missense (*blue*), frameshift/in-frame (*green*) and splicing site (*purple*) mutations were identified.

**Figure 2:**
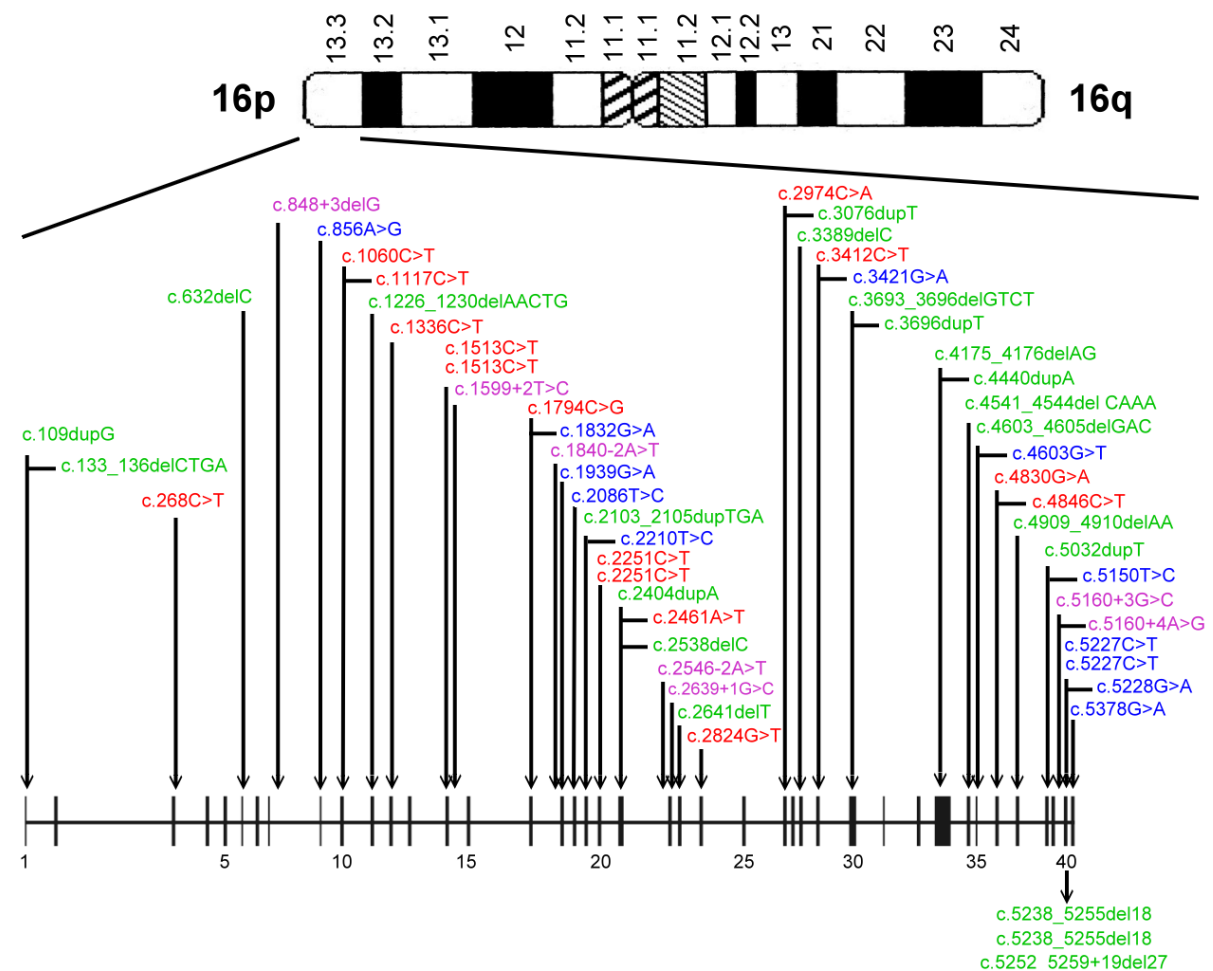
Diagram depicting the locations of mutations in the *TSC2 *gene. Nonsense (*red*), missense (*blue*), frameshift/in-frame (*green*) and splicing site (*purple*) mutations were identified.

Mutations were identified and located in exons of both *TSC1 *and *TSC2 *genes (see Figure 1 and 2). Of the 64 mutations found, nine and 55 were associated with *TSC1 *(14%) and *TSC2 *(86%), respectively, as shown in Table 4. Of the 10 familial cases, 2 (20%) and 8 (80%) were *TSC1*and *TSC2 *mutations, respectively. Among the 54 sporadic cases, 7 *TSC1 *(13%) and 47 *TSC2 *(87%) mutations were found. Accordingly, there was no significant difference between sporadic and familial TSC cases with respect to the frequency of *TSC1 *vs *TSC2 *mutation (*P *= 0.62).

**Table 4:** Distribution of *TSC1 *and *TSC2 *mutations.

	**N**	**MM**	**NM**	**FM**	**SM**	**Total**
***TSC1 *mutaions**						
**Familial**	2	0	1	1	0	2 (3 %)
**Sporadic**	7	0	4	3	0	7 (11 %)
**Total**	9	0 (0 %)	5 (8 %)	4 (6 %)	0 (0 %)	9 (14 %)
***TSC2 *mutations**						
**Familial**	8	4	0	4	0	8 (13 %)
**Sporadic**	47	8	15	17	7	47 (73 %)
**Total**	55	12 (19 %)	15 (23 %)	21 (33 %)	7 (11 %)	55 (86 %)

N: screening numbers.

MM: missense mutations.

NM: nonsense mutations.

FM: frameshift/in-frame mutations.

SM: splicing site mutations.

### Genotype-Phenotype Correlation: Clinical Manifestations

The clinical characteristics associated with each mutation in the proband are shown in Tables 5 (eight *TSC1 *mutations) and Table 6 (43 *TSC2 *mutations). Most patients with *TSC1 *and *TSC2 *mutations had seizures, brain lesions (subependymal nodules and/or cortical tubers detected by MRI), and dermal manifestations. Our criteria for intellectual disability included any degree of mental retardation and learning disorder. The incidence of intellectual disability appeared lower in patients with *TSC1 *mutations (3/8 = 38%) compared to that of patients with *TSC2 *mutations (27/43 = 63%). However, this difference was not statistically significant (*P *= 0.25), but this would be expected because of such small sample sizes. Similarly, the incidence of mental retardation in patients with *TSC1 *mutations (1/8 = 13%) appeared to be less than that of patients with *TSC2 *mutations (17/43 = 40%), but this difference was not statistically significant (*P *= 0.23). Similarly, the frequencies of renal findings, cortical tubers, subependymal giant cell astrocytomas, liver tumors, cardiac tumors, or skin manifestations, including hypomelanotic macules, facial angiofibromas, shagreen patches, and ungual fibromas did not significantly differ between the patients with *TSC1 *and *TSC2 *mutations. However, all of these comparisons are under-powered due to the relatively small number of patients with *TSC1 *mutations that were studied. For nearly all of the clinical features studied, the frequencies were less for those bearing *TSC1 *mutations than for those bearing *TSC2 *mutations. This is consistent with findings from other large studies, showing that *TSC1 *disease is less severe than *TSC2 *disease [9,10,16].

**Table 5:** Clinical data of patients with *TSC1 *mutations

**Family no**.	**Familial/Sporadic**	**Mutation type**	**Sex**	**Onset age of seizure**	**Intellectual performance**	**Brain tubers**	**Renal tumors**	**Hepatic tumors**	**Cardiac rhabdomyoma**	**Hypomelanotic macules**	**Facial angiofibroma**	**Shagreen patch**	**Ungual fibroma**
2	F	FS	F	2 y	N	+	0	NA	NA	0	+	0	+
3	S	NM	M	8 y 3 m	N	+	0	0	0	+	+	+	0
41	S	NM	F	1 y	N	+	0	0	+	+	0	0	0
31	S	NM	F	6 m	LD	+	+	0	+	+	0	0	0
36	S	FS	M	2 y	N	+	0	0	0	+	+	0	0
61	F	NM	M	3 y 6 m	LD	+	0	0	0	+	+	0	0
62	S	FS	M	1 m	LD	+	+	0	0	+	+	+	+
72	S	FS	M	3 y	N	+	0	0	+	+	0	+	0

N: normal or no seizure, LD: learning disorder, MR: metal retardation, NA: not available.

**Table 6:** Clinical data of patients with *TSC2 *mutations

**Family no**.	**Familial/Sporadic**	**Mutation type**	**Sex**	**Onset age of seizure**	**Intellectual performance**	**Brain tubers**	**Renal tumors**	**Hepatic tumors**	**Cardiac rhabdomyoma**	**Hypomelanotic macules**	**Facial angiofibroma**	**Shagreen patch**	**Ungual fibroma**
4	S	MM	M	10 m	N	+	+	0	0	+	+	+	0
5	S	MM	F	1 y	N	+	NA	NA	0	+	+	0	0
6	S	FS	M	1 m	LD	+	0	0	+	+	0	0	0
7	S	MM	M	6 m	MR	NA	NA	NA	NA	+	+	0	0
8	S	S	F	3 m	LD	+	+	0	0	+	+	+	0
9	S	FS	M	5 y	LD	+	0	0	0	+	+	+	0
10	S	FS	F	6 m	LD	+	0	0	+	+	+	0	0
11	F	FS	F	4 m	LD	+	+	0	+	0	+	+	0
12	S	NM	M	10 m	N	+	+	0	NA	+	+	+	0
13	S	FS	F	4 m	MR	+	NA	NA	+	0	0	0	0
15	F	MM	F	7 m	MR	+	0	0	0	+	0	0	0
16	S	FS	M	1 y	N	+	+	3	NA	+	+	+	+
18	S	FS	F	5 m	LD	+	NA	NA	NA	+	+	+	0
19	S	NM	M	3 m	MR	+	+	0	NA	+	0	0	0
20	S	NM	M	1 y 6 m	N	+	+	0	0	+	+	0	0
21	F	FS	F	9 y	N	+	+	1	0	+	+	+	+
22	F	FS	M	1 y	LD	NA	NA	NA	NA	+	+	+	0
23	S	NM	F	7 m	MR	+	NA	NA	NA	+	0	0	0
25	S	FS	M	6 m	LD	+	0	NA	0	+	+	+	0
26	S	FS	F	8 m	LD	+	+	0	0	+	+	+	0
27	S	NM	M	1 y	MR	+	+	0	0	+	+	0	0
28	S	NM	M	3 m	LD	+	NA	NA	NA	+	+	+	0
29	S	MM	F	6 m	MR	+	0	NA	0	+	0	+	0
30	S	FS	F	1 y	MR	+	+	0	0	+	+	0	0
32	S	NM	M	3 m	N	+	+	+	0	+	+	0	0
33	S	FS	F	1 m	N	+	+	+	0	+	+	+	+
34	S	MM	F	7 y	N	+	+	0	0	+	0	+	0
35	S	NM	M	1 y	MR	+	+	0	+	+	+	+	0
37	F	MM	M	9 m	MR	+	0	0	+	+	+	+	0
39	F	FS	F	3 m	MR	+	NA	NA	NA	+	+	+	0
42	F	MM	M	7 y	N	0	+	+	NA	+	+	+	+
47	S	FS	F	2 m	N	+	0	0	+	+	0	0	0
48	S	FS	F	3 m	MR	+	+	0	0	+	+	+	0
50	S	MM	M	3 m	MR	+	NA	NA	NA	+	+	+	0
53	S	FS	M	3 y	MR	+	+	0	0	+	+	+	0
56	F	MM	F	2 y	N	+	+	+	0	+	+	+	+
57	S	NM	F	6 m	MR	+	0	0	0	+	+	+	0
59	S	S	F	1 m	MR	+	+	0	0	+	+	0	0
64	S	NM	M	2 m	N	+	0	0	0	+	0	0	0
67	S	S	F	3 m	MR	+	0	0	+	+	+	0	0
73	S	S	F	21 y	N	+	0	0	0	+	+	0	+
75	S	NM	F	1 y	N	+	+	0	0	+	+	0	0
82	S	MM	M	1 m	N	0	0	0	+	0	0	0	0

N: normal or no seizure, LD: learning disorder, MR: metal retardation, NA: not available.

## Conclusion

This study is the first analysis of *TSC1 *and *TSC2 *genes in the Taiwanese population. We identified 64 mutations among a total of 84 patients (76%); 9 were *TSC1 *mutations (14%) and 55 were *TSC2 *mutations (86%). These numbers are similar to other studies with larger cohorts [9,10,16-18] and would be expected if the germ line mutation rate at the *TSC2 *locus were higher than that at the *TSC1 *locus. The failure to detect mutations in the remaining 24% of the patients may be due to a combination of lack of screening for large genomic deletion and rearrangement mutations in either *TSC1 *or *TSC2*. The occurrence of mosaic mutations [19,20] in some of these patients that may be difficult to detect. Another reason is mutation detection failure.

According to previous reports, somatic and general mosaicism are seen in 6%-10% of all TSC patients [20,21]. In addition, large deletions have been identified in about 2%-4% of *TSC2 *mutations [6] and less commonly in the *TSC1 *gene [22,23]. Thus, both of these situations likely contributed to patients in which mutations were not identified.

In summary, sixty-four different mutations were identified and characterized for the Taiwanese population. Of those, 31 were not previously described. The diverse mutation spectrum of TSC was also seen in different families and different populations.

## Abbreviations

DHPLC: Denaturing high performance liquid chromatography

TSC: Tuberous sclerosis complex

CT: Computed tomography

MRI: Magnetic-resonance imaging

PCR: Polymerase chain reaction

Tm: Melting temperature

## Competing interests

We received financial support in the form of a grant from the National Science Council of Taiwan (NSC 92-2314-B-002-319). We have no other competing interests to declare.

## Authors' contributions

*CCH *and *YNS *performed the molecular genetics studies and drafted the manuscript. *SCC *participated in the molecular genetics studies. *HHL *and *CCC *performed the clinical characterization of the patients. *PCC *and *CJH *performed the statistical analyses. *CPC*, *WTL *and *WLL *participated in the design of the study. *CNL *conceived the study, participated in its design and coordination, and helped draft the manuscript. All authors read and approved the final manuscript.

## Pre-publication history

The pre-publication history for this paper can be accessed here:


